# Clinical Impact and Risk Factors of Seizure After Liver Transplantation: A Nested Case-Control Study

**DOI:** 10.3389/ti.2024.12342

**Published:** 2024-02-27

**Authors:** Minyu Kang, Hwa-Hee Koh, Deok-Gie Kim, Seung Hyuk Yim, Mun Chae Choi, Eun-Ki Min, Jae Geun Lee, Myoung Soo Kim, Dong Jin Joo

**Affiliations:** Department of Surgery, The Research Institute for Transplantation, Yonsei University College of Medicine, Seoul, Republic of Korea

**Keywords:** seizure, liver transplantation, hyponatremia, sodium, neurologic complication

## Abstract

Seizures are a frequent neurological consequence following liver transplantation (LT), however, research on their clinical impact and risk factors is lacking. Using a nested case-control design, patients diagnosed with seizures (seizure group) within 1-year post-transplantation were matched to controls who had not experienced seizures until the corresponding time points at a 1:5 ratio to perform survival and risk factor analyses. Seizures developed in 61 of 1,243 patients (4.9%) at median of 11 days after LT. Five-year graft survival was significantly lower in the seizure group than in the controls (50.6% vs. 78.2%, respectively, *p* < 0.001) and seizure was a significant risk factor for graft loss after adjusting for variables (HR 2.04, 95% CI 1.24–3.33). In multivariable logistic regression, body mass index <23 kg/m^2^, donor age ≥45 years, intraoperative continuous renal replacement therapy and delta sodium level ≥4 mmol/L emerged as independent risk factors for post-LT seizure. Delta sodium level ≥4 mmol/L was associated with seizures, regardless of the severity of preoperative hyponatremia. Identifying and controlling those risk factors are required to prevent post-LT seizures which could result in worse graft outcome.

## Graphical Abstract

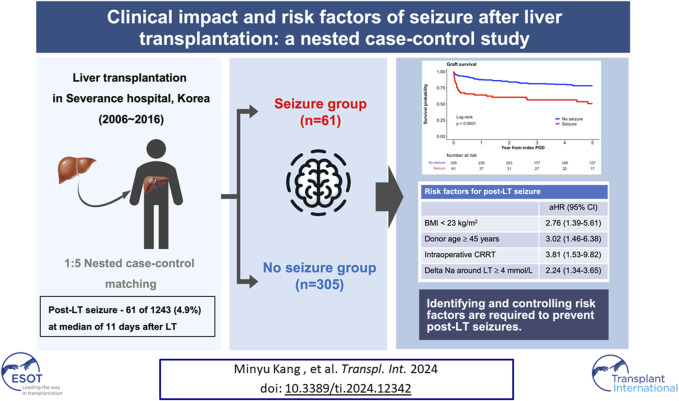

## Introduction

Liver transplantation (LT) is a life-saving procedure for various end-stage liver diseases and hepatocellular carcinoma. Despite significant advancements in surgical techniques and postoperative care, LT patients are susceptible to a range of complications, with neurological events being particularly concerning [1, 2]. Among these, seizures stand out both for their frequency and their impact on patient outcomes [3, 4].

Seizures occur in approximately 10% of LT recipients, a rate notably higher than in other postoperative scenarios [5]. The etiology of these seizures is multifactorial, often involving systemic infections and rapid shifts in electrolyte and osmotic balances. In some instances, seizures are secondary to other neurological events like ischemic strokes or brain hemorrhages [5]. Notably, while demyelinating osmotic syndrome (DOS) and other brain imaging abnormalities can accompany these seizures, they can also occur with no apparent imaging anomalies.

This higher incidence of seizures in LT patients can be attributed to various factors inherent to the transplantation process. Pre-existing conditions like hepatic encephalopathy and hyponatremia in LT candidates have been recognized as contributing factors [6, 7]. Post-transplant, the complex interplay of immunosuppressive therapy, particularly with the widespread use of tacrolimus, infection, and metabolic disturbances, creates a conducive environment for neurological complications [7].

The impact of seizures on LT outcomes has not been extensively studied, especially in the context of long-term survival. Existing studies, albeit limited in volume, indicate a significant association between post-transplant seizures and early mortality [8–10]. However, the long-term implications of these seizures and their specific risk factors remain inadequately explored. This retrospective study aimed to determine the clinical impact and risk factors of seizures after LT.

## Materials and Methods

### Study Population

Among 1,385 LTs performed between July 2005 and December 2021 at Severance Hospital, Korea, where living donor LT is predominant [11]. Patients aged <18 years (*n* = 120), those with a history of seizures before LT (*n* = 15), and those with missing data (*n* = 7) were excluded from the study. From the 1,243 eligible patients who underwent LT, those diagnosed with seizures at 1-year post-transplantation upon consultation with a neurologist were recruited. The causes of seizures were identified using blood tests and brain imaging studies and categorized according to the presence and type of abnormalities in imaging studies.

### Nested Case-Control Design

**FIGURE 1:**
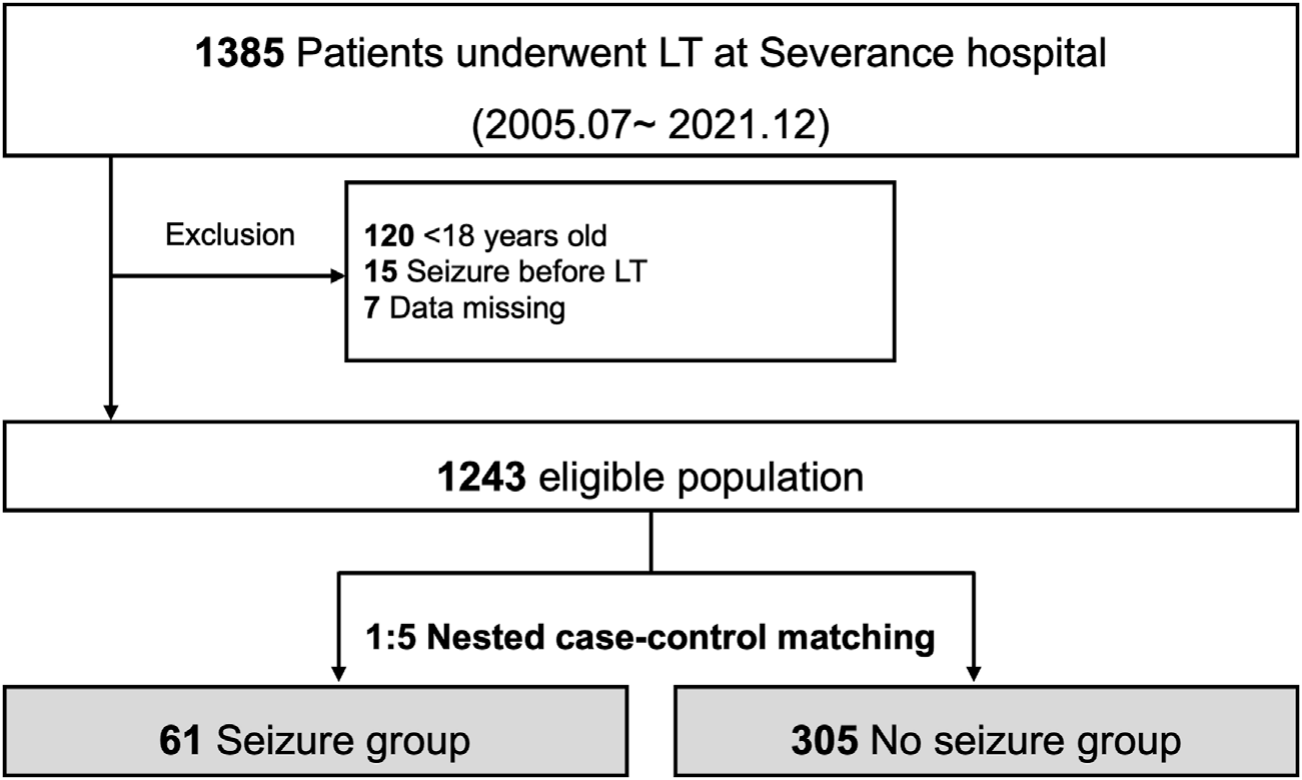
Study flow for nested case-control study.

Patients diagnosed with seizures (seizure group) were matched to controls (no-seizure group) at a 1:5 ratio using a nested case-control design (Figure 1). Possible control individuals who had not experienced seizures (regardless of the possibility of seizures in the future) were randomly sampled at the corresponding time point (index postoperative day [POD]) when seizures developed in the seizure group. The year of LT was matched during the sampling process to ensure a comparable follow-up duration. Patients selected for the no-seizure group at certain time points were reused as potential controls at the subsequent sampling time for the seizure group unless seizures had not occurred before then. These procedures were conducted with *ccwc* function of *Epi* package (version 2.47.1) in R. The resulting left-truncated data were followed from the index POD until death, retransplantation, 5 years after sampling, or June 2022, whichever came first. If the sampled controls experienced seizures thereafter, they were censored during seizure development for the survival analyses.

### Data Collection

Baseline characteristics of the recipient and donor and transplant factors were retrieved from the institutional LT database. In addition, data on pretransplant cerebrovascular accident (CVA), pretransplant dialysis, and intraoperative continuous renal replacement therapy (CRRT) among the sampled cohort were collected from electronic medical records. Pretransplant sodium (Na) level was categorized according to severity, and delta Na level was calculated as the difference between Na levels measured closest to the time of surgery among those recorded before and after the LT.

After applying a nested case-control design, various laboratory results at the index POD of each patient were merged with those of a matched cohort. In addition, the use of each immunosuppressant was defined as prescriptions over 50% of post-transplant days before the index POD. To analyze the association between tacrolimus exposure and seizures, tacrolimus trough levels before the index POD were estimated. Data regarding surgical complications, rejection, and sepsis before the index POD were also collected. Graft loss was defined as patient death or retransplantation.

### Statistical Analysis

According to their normality, data were presented as a number (percentage) for categorical variables and as a median (interquartile range [IQR]) for continuous variables. The chi-square test or Wilcoxon rank-sum test was used, as necessary, to compare the seizure group with the control group. Graft survival following the index POD was compared between the two groups using the Kaplan–Meier curve and log-rank test to examine the clinical impact of seizures. The relationship between seizures and graft survival was assessed using univariate and multivariate Cox regression analyses. For more robustness, graft survival was compared between matched population upon deciles of propensity score (PS) which was calculated using all baseline variables [12]. The matching was considered adequately balanced when the standardized mean differences between the groups were below 0.1 [13].

Risk factors for posttransplant seizures were examined using logistic regression analysis. Significant continuous variables were entered into the model after categorization with cutoff values determined by the Yuden Index, which were analyzed using the pROC package of R software [14, 15]. Considering the relatively small number of events compared to the number of variables we intended to evaluate, multivariable logistic and Cox regression models were created using the backward stepwise method. All analyses were performed using the R statistical package, version 4.2.0 for macOS,^1^ with the threshold for significance set at *p* < 0.05.

### Ethic Approval

This study was performed in accordance with the Declaration of Helsinki and Declaration of Istanbul and was approved by the Institutional Review Board at Severance Hospital, Yonsei University Health System (IRB No. 4-2023-1567). Informed consent was not required because of the study’s retrospective design.

## Results

Of the 1,243 eligible patients, 61 (4.9%) experienced seizures within 1 year after LT (Figure 1). The median time from LT to seizure was 11 (IQR: 6–26) days, and 47 of the 61 (77.0%) patients developed seizures within 30 days after LT (Supplementary Figure S1). Among the 61 patients with seizures, 14 (22.9%) showed structural abnormalities on brain imaging. The brain structural abnormalities identified included cerebral hemorrhage (*n* = 3), cerebral infarction (*n* = 5), posterior reversible encephalopathy syndrome (*n* = 3), DOS (*n* = 2), and hypoxic brain damage (*n* = 1). In the nested case-control design, 305 control patients were matched with the seizure group patients.

### Baseline Characteristics

As shown in Table 1, the age and sex were similar in the seizure and non-seizure groups. The seizure group showed lower BMI than did the control group (22.6 [IQR: 20.7–24.5] kg/m^2^ vs. 23.9 [IQR: 22.0–26.3] kg/m^2^, *p* = 0.007). There were no differences in the incidence of hypertension, diabetes mellitus, or cardiovascular disease between the groups. The frequency of the underlying liver diseases was not statistically different between the groups; however, the seizure group had a higher proportion of patients with alcoholic liver disease than did the control group (39.3% vs. 24.6%). Pretransplant model for end-stage liver disease (MELD) score (23 [IQR: 15–32] vs. 15 [IQR: 10–24], *p* < 0.001) was higher, and pretransplant intensive care unit (ICU) stay (18.0% vs. 4.6%, *p* < 0.001) was more frequent in the seizure group than in the control group. Encephalopathy before LT was more severe in the seizure group than in the control group (*p* = 0.001). The seizure group showed a higher proportion of deceased donor LT (49.2% vs. 31.5%, *p* = 0.005) and an advanced donor age (47 [IQR: 32–54] years vs. 37 [IQR: 27–47] years, *p* = 0.005) than did the control group. The seizure group received more red blood cell transfusion (2.7 [IQR: 1.2–4.5] vs. 1.2 [IQR: 0.6–2.4] L, *p* < 0.001), had more pretransplant CVAs (6.6% vs. 1.0%, *p* = 0.017), and received more pretransplant dialysis (23.0% vs. 7.5%, *p* = 0.001) and intraoperative CRRT (36.1% vs. 7.5%, *p* < 0.001) than did the control group. Severe pretransplant hyponatremia was observed in the seizure group (9.8% vs. 2.6%, *p* = 0.007), and delta Na (5 [IQR: 4–7] vs. 3 [IQR: 2–6], *p* < 0.001) was also higher in the seizure group than in the control group.

**TABLE 1:** Baseline characteristics.

Variables	Seizure (*n* = 61)	No seizure (*n* = 305)	*P*
Age	53 (45–63)	54 (47–59)	0.941
Sex, female	13 (21.3)	94 (30.8)	0.181
BMI, kg/m^2^	22.6 (20.7–24.5)	23.9 (22.0–26.3)	0.007
Year of LT			1.000
2012–2015	27 (44.3)	135 (44.3)	
2016–2018	21 (34.4)	105 (34.4)	
2019–2021	13 (21.3)	65 (21.3)	
Hypertension	9 (14.8)	75 (24.6)	0.133
Diabetes mellitus	17 (27.9)	88 (28.9)	1.000
Cardiovascular disease	8 (13.1)	25 (8.2)	0.327
Underlying liver disease			0.084
Viral	32 (52.5)	172 (56.4)	
Alcoholic	24 (39.3)	75 (24.6)	
Others	5 (8.2)	58 (19.0)	
HCC	25 (41.0)	149 (48.9)	0.326
Pretransplant MELD	23 (15–32)	15 (10–24)	<0.001
Pretransplant stay			<0.001
Out-patient day	24 (39.3)	161 (52.8)	
Ward	26 (42.6)	130 (42.6)	
Intensive care unit	11 (18.0)	14 (4.6)	
Refractory ascites	20 (32.8)	54 (17.7)	0.012
Encephalopathy			0.001
No	32 (52.5)	231 (75.7)	
Mild	20 (32.8)	49 (16.1)	
Moderate to severe	9 (14.8)	25 (8.2)	
Re-transplantation	1 (1.6)	6 (2.0)	1.000
ABO incompatibility	5 (8.2)	51 (16.7)	0.135
Donor type			0.012
Living	31 (50.8)	209 (68.5)	
Deceased	30 (49.2)	96 (31.5)	
Donor age	47 (32–54)	37 (27–47)	0.005
Donor sex, female	14 (23.0)	104 (34.1)	0.121
Donor BMI	22.3 (20.5–24.7)	22.9 (21.1–24.7)	0.277
Operation time, min	594 (472–660)	592 (504–699)	0.284
RBC transfusion, L	2.7 (1.2–4.5)	1.2 (0.6–2.4)	<0.001
Pretransplant CVA	4 (6.6)	3 (1.0)	0.017
Pretransplant dialysis	14 (23.0)	23 (7.5)	0.001
Intraoperative CRRT	22 (36.1)	23 (7.5)	<0.001
Pretransplant hyponatremia			0.007
Normal (≥135 mmol/L)	39 (63.9)	242 (79.3)	
Mild (130–134 mmol/L)	13 (21.3)	36 (11.8)	
Moderate (126–129 mmol/L)	3 (4.9)	19 (6.2)	
Severe (<126 mmol/L)	6 (9.8)	8 (2.6)	
Delta Na around LT^a^, mmol/L	5 (4–7)	3 (2–6)	<0.001

BMI, body mass index; CRRT, continuous renal replacement therapy; CVA, cerebrovascular accident; HCC, hepatocellular carcinoma; LT, liver transplantation; MELD, model for end-stage liver disease; RBC, red blood cell.

^a^ Difference of Na between before and after LT, within 24 h.

### Information at Index POD

At matched index POD, the seizure group showed higher total bilirubin (3.1 [IQR: 1.1–8.3] mg/dL vs. 1.3 [IQR: 0.8–2.6] mg/dL, *p* < 0.001), blood urea nitrogen (27.8 [IQR: 18.6–47.3] mg/dL vs. 20.3 [IQR: 14.5–31.7] mg/dL, *p* < 0.001), and glucose (156 [IQR: 134–184] mg/dL vs. 138 [IQR: 113–181] mg/dL, *p* = 0.014, Table 2) levels than did the control group. Albumin level was lower in the seizure group than in the control group (3.1 [IQR: 2.9–34] mg/dL vs. 3.4 [IQR: 3.1–3.7] mg/dL, *p* < 0.001). Hemoglobin level (9.5 [IQR 8.3–10.5] g/dL vs. 10.1 [IQR 8.9–11.3] g/dL, *p* = 0.004) and platelet count (63 [IQR 45–126] × 10^3^/μL vs. 110 [67–165] × 10^3^/μL, *p* < 0.001) were also lower in the seizure group than in the control group. The use of each immunosuppressant and tacrolimus trough level were similar between the groups in terms of the mean, standard deviation, maximum variance, and coefficient of variance. The rates of post-LT complications, such as rejection, bile duct complications, vascular complications, and reoperation, prior to the index POD were also similar between the groups, except for sepsis, which was higher in the seizure group than in the control group (16.4% vs. 6.9%, *p* = 0.029).

**TABLE 2:** Information at index POD.

Variables^a^	Seizure (*n* = 61)	No seizure (*n* = 305)	P
Total bilirubin, mg/dL	3.1 (1.1–8.3)	1.3 (0.8–2.6)	<0.001
AST, IU/L	43 (27–79)	33 (21–74)	0.110
ALT, IU/L	54 (21–79)	57 (20–133)	0.274
Creatinine, mg/dL	0.9 (0.6–1.4)	0.8 (0.6–1.2)	0.269
BUN, mg/dL	27.8 (18.6–47.3)	20.3 (14.5–31.7)	<0.001
Albumin, mg/dL	3.1 (2.9–3.4)	3.4 (3.1–3.7)	<0.001
Glucose, mg/dL	156 (134–184)	138 (113–181)	0.014
Na, mmol/L	138 (135–142)	138 (136–140)	0.955
White blood cell, 10^3^/μL	6.9 (4.5–12.1)	6.2 (4.6–9.0)	0.171
Hemoglobin, g/dL	9.5 (8.3–10.5)	10.1 (8.9–11.3)	0.004
Platelet, 10^3^/μL	63 (45–126)	110 (67–165)	<0.001
Use of immunosuppressants^b^			
Tacrolimus	60 (98.4)	298 (97.7)	1.000
Mycophenolate mofetil	23 (37.7)	150 (49.2)	0.134
mTOR inhibitor	4 (6.6)	17 (5.6)	1.000
Steroid	53 (86.9)	269 (88.2)	0.943
Tacrolimus trough level, ng/dL^b^			
Mean	6.6 (4.5–8.7)	7.3 (5.4–10.3)	0.100
Standard deviation	2.9 (2.1–4.3)	2.5 (1.5–3.9)	0.287
Maximum	11.1 (7.7–18.0)	11.3 (7.8–17.8)	0.826
Variance	7.7 (5.5–11.0)	8.0 (5.5–12.7)	0.214
Coefficient of variance	8.5 (4.3–18.9)	6.4 (2.2–15.4)	0.287
Prior rejection	3 (4.9)	34 (11.1)	0.215
Prior bile duct complication	4 (6.6)	22 (7.2)	1.000
Prior vascular complication	4 (6.6)	5 (1.6)	0.070
Reoperation	14 (23.0)	59 (19.3)	0.640
Sepsis	10 (16.4)	21 (6.9)	0.029

ALT, alanine aminotransferase; AST, aspartate aminotransferase; BUN, blood urea nitrogen; mTOR, mammalian target of rapamycin; POD, post-operative day.

^a^ Values were acquired from LT to index POD in each patients.

^b^ Use of each immunsuppressants was defined as prescription at over 50% of post-transplant days before index POD.

### Seizure and Graft Survival

**FIGURE 2:**
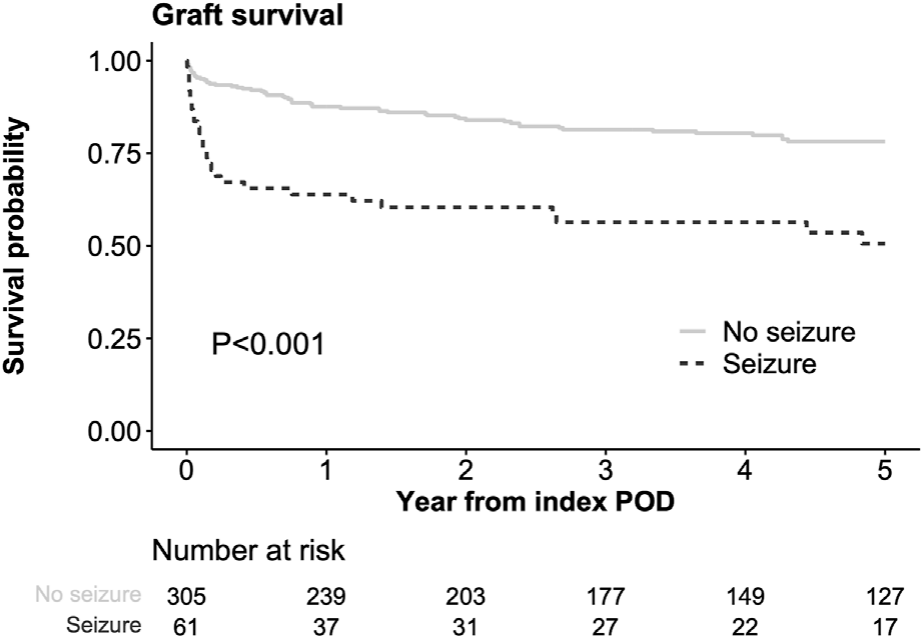
Comparison of graft survival from index POD Index POD was the time of seizure occurrence in the seizure group and the corresponding POD in the matched control groups.

Among 366 matched population, 86 patients (23.5%) experienced graft loss (85 death and 1 retransplantation) within 5 years after index POD. In the Kaplan-Meier analysis, graft survival rate after the index POD was significantly lower in the seizure group than in the control group (63.9%, 56.4%, and 50.6% at 1, 3, and 5 years, respectively, in the seizure group vs. 87.5%, 81.4%, and 78.2% at 1, 3, and 5 years, respectively, in the no-seizure group, *p* < 0.001, Figure 2). In uni- and multivariable Cox regression models, post-LT seizure was an independent risk factor for graft loss in the matched cohort (hazard ratio [HR]: 2.04, 95% CI: 1.24–3.33, Supplementary Table S1). When the seizure group was matched with those who did not experience seizure on PS, hazardous effect of seizure on the graft survival was also observed (Supplementary Figure S2), although all variables were balanced between two groups (Supplementary Table S2).

Infection, graft failure, and HCC recurrence were three most common causes of death in both groups (Supplementary Figure S3). Among them, infectious death was significantly higher in the seizure group than the control group (18.0% vs. 5.9%, *p* = 0.003, Supplementary Table S3).

### Risk Factors for Seizure After LT

In uni- and multivariable logistic regressions (Table 3), the independent risk factors for post-LT seizures were BMI <23 kg/m^2^ (odds ratio [OR]: 2.76, 95% CI: 1.39–5.61), donor age ≥45 years (OR: 3.02, 95% CI: 1.46–6.38), intraoperative CRRT (OR: 3.81, 95% CI: 1.53–9.82), and delta Na ≥4 mmol/L (OR: 5.38, 95% CI: 2.55–12.3). Among laboratory values at index POD, total bilirubin level ≥2.5 mg/dL (OR: 2.50, 95% CI: 1.21–5.24) and albumin level <3.5 mg/dL (OR: 6.75, 95% CI: 2.13–28.4) emerged as independent risk factors for post-LT seizures. When we performed sensitivity analysis only including seizures without structural abnormality, same risk factors were observed (Supplementary Table S4).

**TABLE 3:** Risk factor analyses for seizure after LT.

Variables	Univariable		Multivariable^a^	
	OR (95% CI)	*P*	OR (95% CI)	*P*
BMI < 23 kg/m^2^	2.13 (1.23–3.75)	0.008	2.76 (1.39–5.61)	0.004
Alcoholic	1.99 (1.11–3.52)	0.019	1.77 (0.85–3.66)	0.123
Pretransplant MELD ≥18	2.90 (1.64–5.23)	<0.001	0.72 (0.30–1.70)	0.466
Encephalopathy				
No	—		—	
Mild	2.95 (1.54–5.55)	<0.001	1.48 (0.61–3.46)	0.375
Moderate to severe	2.60 (1.07–5.91)	0.027	1.99 (0.64–6.08)	0.227
Donor age ≥ 45 years	2.91 (1.66–5.12)	<0.001	3.02 (1.46–6.38)	0.003
Pretransplant CVA	7.06 (1.52–36.6)	<0.001	3.33 (0.63–19.3)	0.155
RBC transfusion ≥ 2L	3.63 (2.06–6.54)	<0.001	1.24 (0.59–2.57)	0.570
Intraoperative CRRT	6.92 (3.52–13.6)	<0.001	3.81 (1.53–9.82)	0.005
Delta Na around LT ≥ 4 mmol/L	3.56 (1.93–6.96)	<0.001	5.38 (2.55–12.3)	<0.001
Laboratory results at index POD				
Total bilirubin ≥ 2.5 mg/dL	4.57 (2.59–8.23)	<0.001	2.50 (1.21–5.24)	0.014
Albumin < 3.5 mg/dL	6.06 (2.40–20.4)	<0.001	6.75 (2.13–28.4)	0.003

BMI, body mass index; CRRT, continuous renal replacement therapy; CVA, cerebrovascular accident; HCC, hepatocellular carcinoma; LT, liver transplantation; MELD, model for end-stage liver disease; RBC, red blood cell.

^a^ Model was established by backward stepwise method and only variables included in the model were presented.

### Delta Na and Seizure Risk in Pre-LT Hyponatremia Subgroups

In the subgroup without pre-LT hyponatremia (≥135 mmol/L), the incidence of seizures was significantly higher when delta Na was ≥4 mmol/L than when it was <4 mmol/L (21.2% vs. 7.4%, *p* = 0.002; Table 4). In the subgroup with pre-LT hyponatremia (<135 mmol/L), post-transplant seizures occurred more frequently when the delta Na was ≥4 mmol/L than when it was <4 mmol/L, although this was not significant owing to the small effect size (30.2% vs. 13.6%, *p* = 0.215). Delta Na ≥4 mmol/L was significantly associated with post-transplant seizure after adjustment of other risk factors in both subgroups with (OR: 5.16, 95% CI: 2.12–14.1) or without pre-LT hyponatremia (OR: 11.2, 95% CI: 1.79–120, Supplementary Table S5).

**TABLE 4:** Incidence of seizure by pretransplant hyponatremia and delta Na around LT.

	PreLT Na ≥ 135 (*n* = 281)			PreLT Na < 135 (*n* = 85)		
	Delta Na ≥ 4 (*n* = 132)	Delta Na < 4 (*n* = 149)	P	Delta Na ≥ 4 (*n* = 63)	Delta Na < 4 (*n* = 22)	P
Group			0.002			0.215
No seizure	104 (78.8%)	138 (92.6%)		44 (69.8%)	19 (86.4%)	
Seizure	28 (21.2%)	11 (7.4%)		19 (30.2%)	3 (13.6%)	

## Discussion

This study demonstrated the clinical impact of post-LT seizures and their associated risk factors using a retrospective nested case-control design. Among the matched population, the incidence of seizure was significantly associated with a low graft survival rate, even after adjusting for baseline covariates and various information at matched time points after LT. Furthermore, we demonstrated various risks factors for seizure including change of Na, which contains clinical implication for the prevention of seizure after LT.

LT candidates often have complications such as hepatic encephalopathy and hyponatremia, and patients with high MELD scores and acute/acute-on-chronic liver failure are often ICU stay- or ventilator-dependent [6, 16–18]. In addition to conditions before LT, various post-LT factors, such as electrolyte alteration, infection, and medications, including CNI, could cause a higher incidence of seizures [5]. Reports of seizures after other non-brain surgeries are fewer than those on the incidence of seizures after cardiac surgery (2.7%); the incidence of seizures in patients who underwent LT is higher than that in patients who underwent other surgeries [19]. Post-LT seizures can cause prolonged ICU stay and ventilator use and can be a critical cause of worsening LT outcomes, regardless of the liver graft function. This could be supported by higher infectious death in the seizure group (18.0%) than in the controls (5.9%) among our study population. Therefore, identifying the risk factors for seizures after LT and managing modifiable factors can improve LT outcomes. This study suggests that management strategies, including limiting excessive Na replacement before and after LT, can prevent LT mortality due to seizures.

Approximately 23% of adult patients who experience their first epileptic seizure reportedly show abnormalities on brain imaging [20]. The prognosis after seizures worsens when structural problems are present [21]. Seizures in patients who underwent LT have also been reported to be accompanied by structural abnormalities such as stroke, DOS, and PRES, but few studies have focused on the seizure itself or examined the proportion of structural abnormalities [2, 5, 22, 23]. In the LT population in this study, seizures occurred without imaging abnormalities in 83.6% of patients with seizures. The overall incidence rate was 4.9%, and most cases (77.0%) occurred within 30 days of surgery. This is thought to be because patients who underwent LT have different risks and mechanisms of seizure occurrence compared with those of the general population. Therefore, it is important to identify LT-specific seizure risk factors and the correct modifiable factors.

The grade of encephalopathy before LT has been reported as an important risk factor for neurological complications in previous studies [6, 24, 25]. Patients with alcoholic liver cirrhosis are prone to Wernicke’s encephalopathy with associated seizures [26, 27]. However, in this study, an increase in Na concentration before and after LT was an important risk factor, regardless of these factors. The occurrence of DOS due to rapid Na correction to 10–12 mmol/L within 24 h in transplant patients with hyponatremia is a well-known complication [22]. However, this study showed that even a mild increase in sodium (≥4 mmol/L) increases the risk of seizures regardless of the severity of pretransplant hyponatremia. More research is required to determine whether focused management of perioperative Na alterations can successfully prevent post-transplant seizures.

A low BMI was an independent risk factor for seizures after LT. Low BMI was considered an indicator of sarcopenia in our LT population, which has been shown to be related to neurological disorders such as dementia, ischemic stroke, depression, and cognitive impairment in recent studies [28]. Dopaminergic dysfunction, neuronal hypoexcitability, brain atrophy, and neuromuscular junction dysfunction are the regulatory processes associated with the pathophysiology of sarcopenia. Although evidence is insufficient, various hormonal and electrophysiological changes in patients with sarcopenia can lead to a high incidence of post-LT seizures [29]. Furthermore, low BMI represents malnutrition which may enhance the tacrolimus induced neurotoxicity [30]. Although sarcopenia and seizure risk have not yet been directly studied, and sarcopenia was not directly measured in this study, its association with neuropsychiatric complications is clear; therefore, it is thought that the control and treatment of sarcopenia to prevent seizures are also important.

Intraoperative CRRT is a strong risk factor for seizures after LT. In contrast to the typical seizure prevalence of 1%, patients with renal failure reported a lifetime seizure prevalence of 9% due to uremia and electrolyte disturbances [31]. Patients receiving hemodialysis are more likely to experience seizures not only because of a higher chance of hypotension and electrolyte imbalance but also because electrolytes are cleared more quickly from their blood than from their cerebral spinal fluid [32]. Because most patients undergoing CRRT have acute or acute-on-chronic liver failure, the underlying cirrhosis itself may be severe, and the seizure risk may be increased. Current hypoalbuminemia was also a significant risk factor for seizures. Hypoalbuminemia also reflects the severity of the liver failure, which is indicated by an MELD score of 3.0 [33]. Nevertheless, the association between albumin level and seizure risk should be evaluated in future studies.

Retrospective features could have resulted in a selection bias between patients who experienced seizures and controls in this study. In addition, the actual type of seizure was not identified, and the difference between seizures with or without imaging abnormalities was not validated owing to the relatively small number of patients in the seizure group. Finally, our results should be interpreted with caution because the living donor LT-dominant population usually undergoes surgery in an elective setting.

Despite these limitations, this nested case-control study demonstrated that seizure occurrence could be related with low graft survival rate. In addition to low BMI, advanced donor age, intraoperative CRRT, total bilirubin and albumin levels, and perioperative Na change ≥4 mmol/L significantly increased post-LT seizures. Identifying and controlling those risk factors may aid in the prevention of post-LT seizures.

## Data Availability

The raw data supporting the conclusion of this article will be made available by the authors, without undue reservation.

## Footnotes

1
http://cran.r-project.org

## Abbreviations

BMI, body mass index; CI, confidence interval; DOS, demyelinating osmotic syndrome; CRRT, continuous renal replacement therapy; CVA, cerebrovascular accident; HR, hazard ratio; IQR, interquartile range; LT, liver transplantation; MELD, model for end-stage liver disease; NA, sodium; OR, odds ratio; POD, postoperative day; PS, propensity score.
