# Supplementary material for: Clinical Impact and Risk Factors of Seizure After Liver Transplantation: A Nested Case-Control Study
Source: Transpl Int. 2024 Feb 27;37:12342. doi: 10.3389/ti.2024.12342 (PMC10930032; doi:10.3389/ti.2024.12342)
Supplement: Supplementary file 1 [file DataSheet1.docx]

**Figure S1. Density plot for the development of seizure.**

(a) within 1 year, (b) within 30 days

**
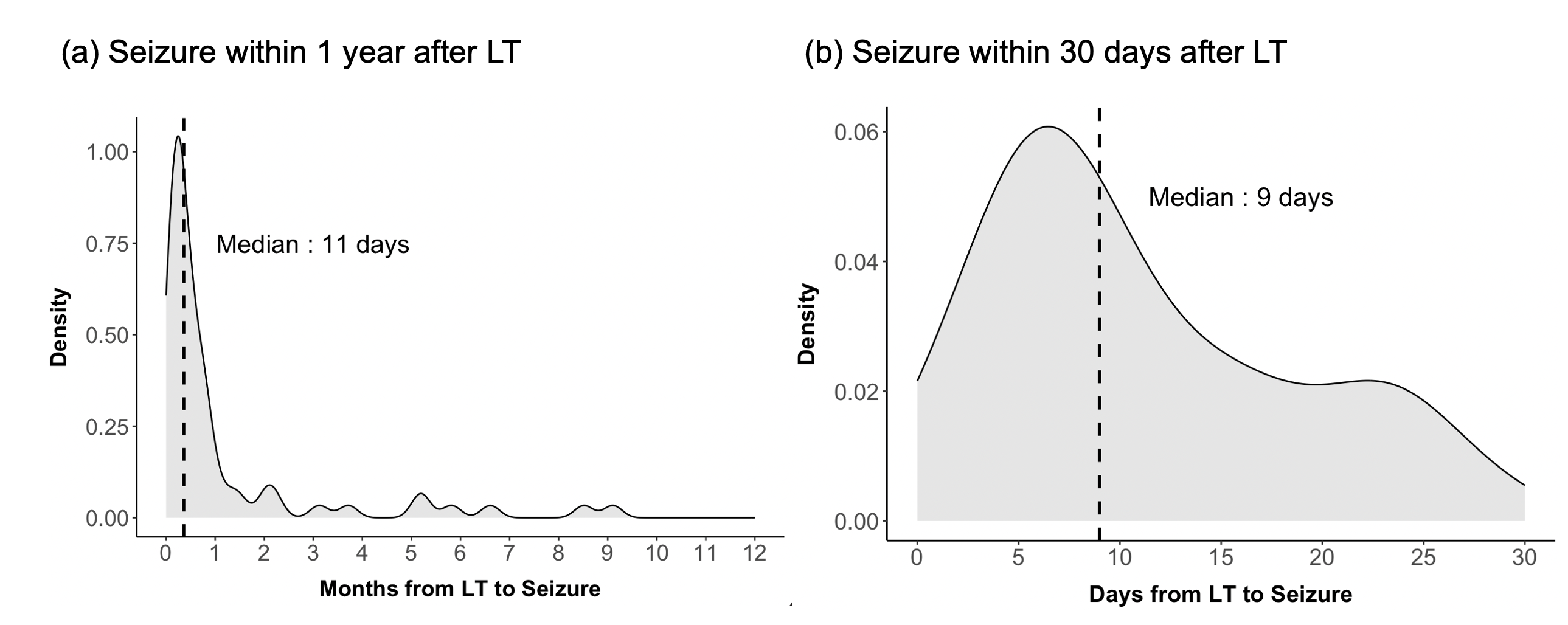
**

**Table S1. Uni- and multivariable Cox regression for graft loss**

**
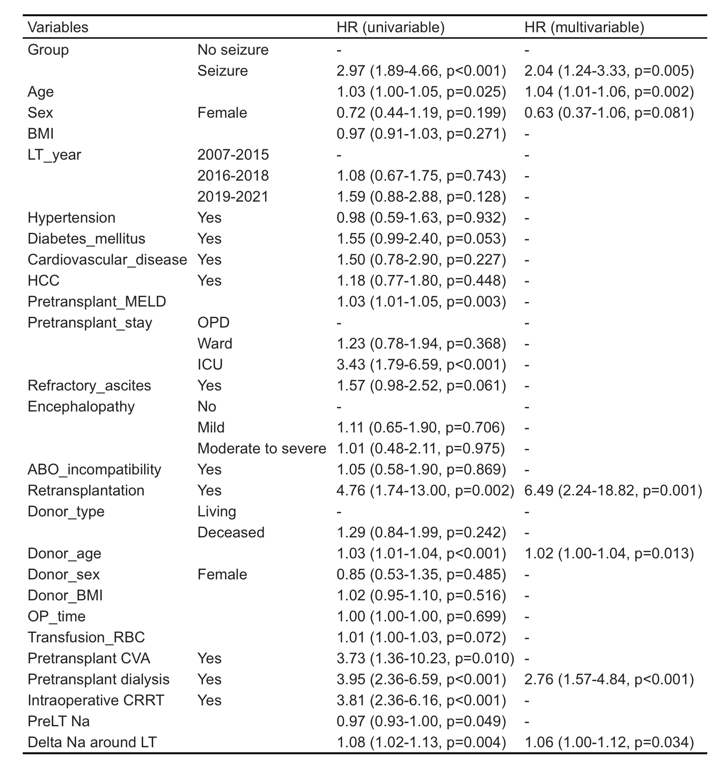
**

**Table S2. Baseline characteristics between PS-matched cohort**

| **Variables** | **Seizure**  **(n = 61)** | **No seizure**  **(n = 305)** | ***P*** |
| --- | --- | --- | --- |
| Age | 53 (45-63) | 54 (48-60) | 0.955 |
| Sex, female | 13 (21.3) | 68 (22.3) | 1.000 |
| BMI, kg/m^2^ | 22.6 (20.7-24.5) | 22.8 (20.6-24.8) | 0.951 |
| Year of LT |  |  | 0.980 |
| 2012-2015 | 27 (44.3) | 133 (43.6) |  |
| 2016-2018 | 21 (34.4) | 109 (35.7) |  |
| 2019-2021 | 13 (21.3) | 63 (20.7) |  |
| Hypertension | 9 (14.8) | 40 (13.1) | 0.891 |
| Diabetes mellitus | 17 (27.9) | 81 (26.6) | 0.958 |
| Cardiovascular disease | 8 (13.1) | 33 (10.8) | 0.767 |
| Underlying liver disease |  |  | 0.898 |
| Viral | 32 (52.5) | 151 (49.5) |  |
| Alcoholic | 24 (39.3) | 125 (41.0) |  |
| Others | 5 (8.2) | 29 (9.5) |  |
| HCC | 25 (41.0) | 117 (38.4) | 0.810 |
| Pretransplant MELD | 23 (15-32) | 22 (13-32) | 0.662 |
| Pretransplant stay |  |  | 0.636 |
| Out-patient day | 24 (39.3) | 138 (45.2) |  |
| Ward | 26 (42.6) | 123 (40.3) |  |
| Intensive care unit | 11 (18.0) | 44 (14.4) |  |
| Refractory ascites | 20 (32.8) | 101 (33.1) | 1.000 |
| Encephalopathy |  |  | 0.759 |
| No | 32 (52.5) | 173 (56.7) |  |
| Mild | 20 (32.8) | 96 (31.5) |  |
| Moderate to severe | 9 (14.8) | 36 (11.8) |  |
| Re-transplantation | 1 (1.6) | 4 (1.3) | 1.000 |
| ABO incompatibility | 5 (8.2) | 23 (7.5) | 1.000 |
| Donor type |  |  | 0.639 |
| Living | 31 (50.8) | 168 (55.1) |  |
| Deceased | 30 (49.2) | 137 (44.9) |  |
| Donor age | 47 (32-54) | 41 (28-53) | 0.492 |
| Donor sex, female | 14 (23.0) | 65 (21.3) | 0.910 |
| Donor BMI | 22.3 (20.5-24.7) | 22.8 (21.1-24.4) | 0.509 |
| Operation time, min | 594 (472-660) | 574 (480-676) | 0.965 |
| RBC transfusion, L | 2.7 (1.2-4.5) | 2.4 (1.3-4.7) | 0.117 |
| Pretransplant CVA | 4 (6.6) | 14 (4.6) | 0.746 |
| Pretransplant dialysis | 14 (23.0) | 58 (19.0) | 0.597 |
| Intraoperative CRRT | 22 (36.1) | 89 (29.2) | 0.360 |
| Pretransplant hyponatremia |  |  | 0.573 |
| Normal (≥ 135 mmol/L) | 39 (63.9) | 184 (60.3) |  |
| Mild (130-134 mmol/L) | 13 (21.3) | 79 (25.9) |  |
| Moderate (126-129 mmol/L) | 3 (4.9) | 23 (7.5) |  |
| Severe (<126 mmol/L) | 6 (9.8) | 19 (6.2) |  |

**Figure S2. Comparison of graft survival between PS-matched population**

**
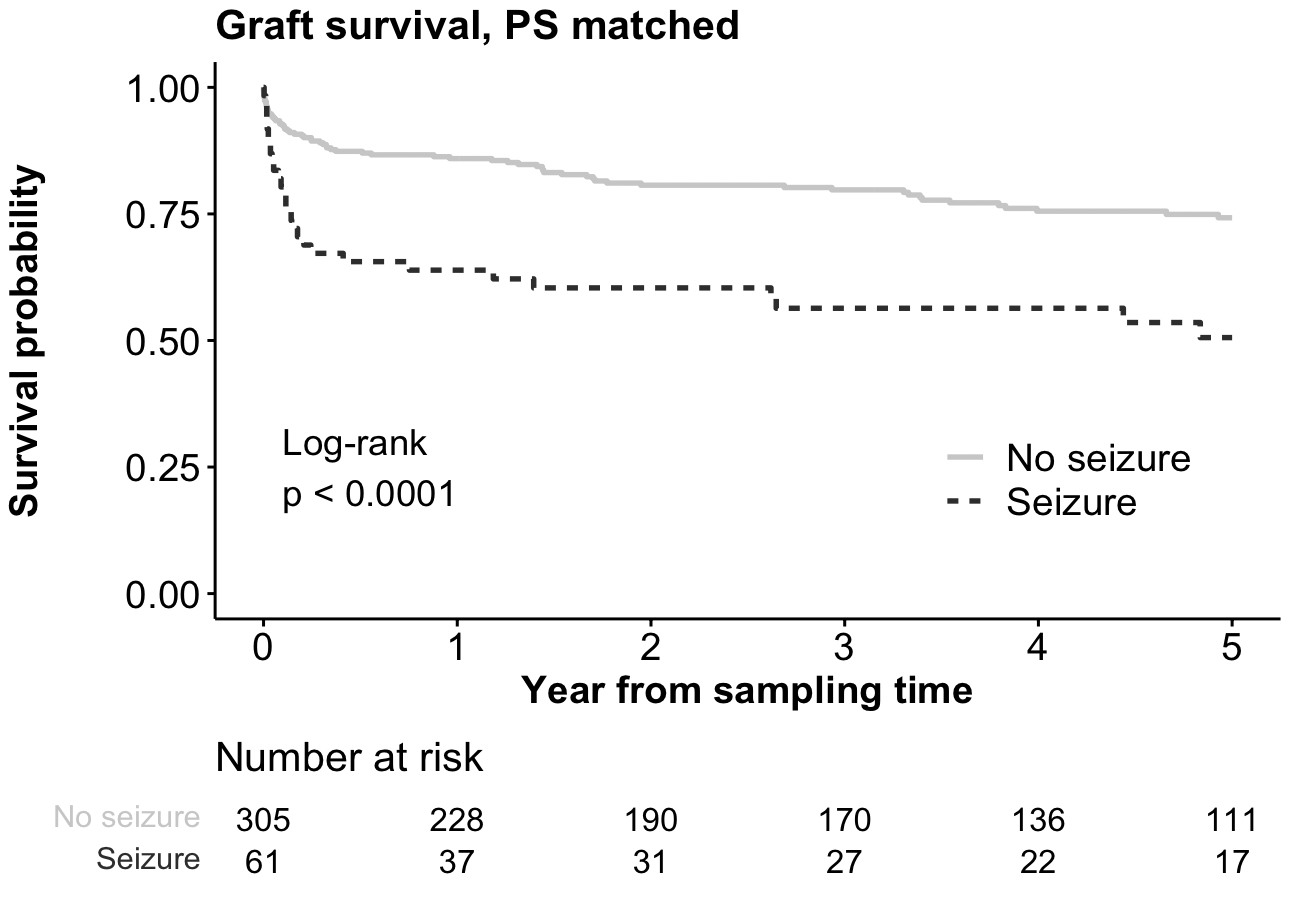
**

**Figure S3. Causes of death**

**
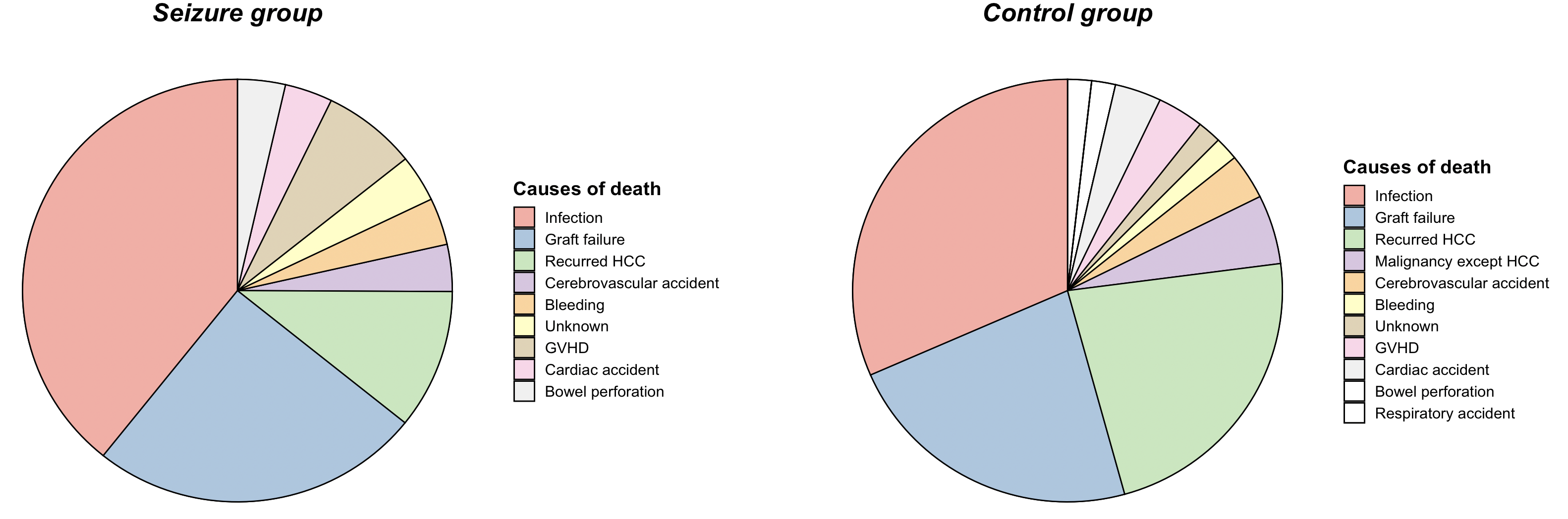
**

**Table S3. Comparison of three main causes of death**

| **Cause of death** | **Seizure**  **(n = 61)** | **No seizure**  **(n = 305)** | ***P*** |
| --- | --- | --- | --- |
| Infection | 11 (18.0%) | 18 (5.9%) | 0.003 |
| Graft failure | 7 (11.5%) | 13 (4.3%) | 0.052 |
| HCC recurrence | 3 (4.9%) | 13 (4.3%) | 0.989 |

**Table S4. Risk factors for seizure only including non-structural causes**

|  | **Multivariable** | |
| --- | --- | --- |
| **Variables** | **OR (95% CI)** | ***P*** |
| BMI < 23 kg/m^2^ | 2.38 (1.24-4.65) | 0.010 |
| Alcoholic | 1.91 (0.95-3.81) | 0.067 |
| Pretransplant MELD ≥18 | 0.97 (0/93-1.01) | 0.141 |
| Donor age ≥ 45 years | 3.33 (1.65-6.86) | <0.001 |
| Intraoperative CRRT | 4.65 (1.71-13.2) | 0.003 |
| Delta Na around LT ≥ 4 mmol/L | 1.12 (1.04-1.22) | 0.005 |
| Laboratory results at index POD |  |  |
| Total bilirubin ≥ 2.5 mg/dL | 3.26 (1.64-6.61) | <0.001 |
| Albumin < 3.5 mg/dL | 5.43 (1.79-22.1) | 0.007 |

**Table S5. Subgroup analyses for seizure risk factors according to pretransplant hyponatremia**

|  | **Pre-LT Na >= 135**  **(n=281)** | |  | **Pre-LT Na < 135**  **(n=85)** | |
| --- | --- | --- | --- | --- | --- |
| **Variables** | **OR (95% CI)** | ***P*** |  | **OR (95% CI)** | ***P*** |
| BMI < 23 kg/m^2^ | 2.37 (1.23-4.67) | 0.011 |  | 1.87 (0.53-6.81) | 0.327 |
| Alcoholic | 1.80 (0.88-3.62) | 0.101 |  | 2.41 (0.57-11.1) | 0.239 |
| Pretransplant MELD ≥18 | 0.81 (0.35-1.83) | 0.617 |  | 0.61 (0.13-2.66) | 0.504 |
| Encephalopathy | 1.56 (0.72-3.35) | 0.258 |  | 0.77 (0.19-3.00) | 0.712 |
| Donor age ≥ 45 years | 1.85 (0.62-4.71) | 0.196 |  | 8.45 (2.14-42.4) | 0.004 |
| Pretransplant CVA | 5.97 (0.93-51.3) | 0.068 |  | - |  |
| RBC transfusion ≥ 2L | 1.16 (0.48-2.76) | 0.732 |  | 3.63 (0.72-21.6) | 0.131 |
| Intraoperative CRRT | 6.37 (1.85-23.9) | 0.004 |  | 0.95 (0.19-4.75) | 0.951 |
| Delta Na around LT ≥ 4 mmol/L | 5.16 (2.12-14.1) | <0.001 |  | 11.2 (1.79-120) | 0.022 |
| Laboratory results at index POD |  |  |  |  |  |
| Total bilirubin ≥ 2.5 mg/dL | 2.46 (0.96-6.46) | 0.063 |  | 8.09 (1.87-44.0) | 0.009 |
| Albumin < 3.5 mg/dL | 4.98 (1.33-25.7) | 0.031 |  | - |  |
